# Influence of doctor-patient conversations on behaviours of patients presenting to primary care with new or persistent symptoms: a video observation study

**DOI:** 10.1136/bmjqs-2019-009485

**Published:** 2019-07-20

**Authors:** Dorothee Amelung, Katriina L Whitaker, Debby Lennard, Margaret Ogden, Jessica Sheringham, Yin Zhou, Fiona M Walter, Hardeep Singh, Charles Vincent, Georgia Black

**Affiliations:** 1 School of Health Sciences, University of Surrey, Guildford, UK; 2 Public Involvement Programme (People in Research), National Institute for Health and Care Excellence, London, UK; 3 Department of Applied Health Research, University College London, London, UK; 4 Primary Care Unit, Department of Public Health and Primary Care, University of Cambridge, Cambridge, UK; 5 Center for Innovations in Quality, Effectiveness and Safety, Michael E. DeBakey Veterans Affairs Medical Center and Baylor College of Medicine, Houston, Texas, USA; 6 Medical Science Division, University of Oxford, Oxford, UK

**Keywords:** primary care, qualitative research, patient safety

## Abstract

**Background:**

Most cancers are diagnosed following contact with primary care. Patients diagnosed with cancer often see their doctor multiple times with potentially relevant symptoms before being referred to see a specialist, suggesting missed opportunities during doctor-patient conversations.

**Objective:**

To understand doctor-patient communication around the significance of persistent or new presenting problems and its potential impact on timely cancer diagnosis.

**Research design:**

Qualitative thematic analysis based on video recordings of doctor-patient consultations in primary care and follow-up interviews with patients and doctors. 80 video observations, 20 patient interviews and 7 doctor interviews across 7 general practices in England.

**Results:**

We found that timeliness of diagnosis may be adversely affected if doctors and patients do not come to an agreement about the presenting problem’s significance. ‘Disagreements’ may involve misaligned cognitive factors such as differences in medical knowledge between doctor and patient or misaligned emotional factors such as patients’ unexpressed fear of diagnostic procedures. Interviews suggested that conversations where the difference in views is either not recognised or stays unresolved may lead to unhelpful patient behaviour after the consultation (eg, non-attendance at specialist appointments), creating potential for diagnostic delay and patient harm.

**Conclusions:**

Our findings highlight how doctor-patient consultations can impact timely diagnosis when patients present with persistent or new problems. Misalignments were common and could go unnoticed, leaving gaps for potential to cause patient harm. These findings have implications for timely diagnosis of cancer and other serious disease because they highlight the complexity and fluidity of the consultation and the subsequent impact on the diagnostic process.

## Introduction

Timely assessment of new and unexplained symptoms is critical in primary care and of particular importance in the case of cancer, where faster diagnosis is an international priority.^1^ Timely diagnosis of cancer largely relies on understanding and improving patients’ pathways to treatment, including shortening the time it takes from noticing a bodily change to consulting a primary care doctor, engaging in onward investigation or referral and planning treatment.^2^ There has been considerable focus on understanding influences on the patient interval (time from detecting a bodily change to first primary care consultation)^3 4^ and more recently, recognition of the importance of the diagnostic interval (time from first consultation in primary care to diagnosis).

Approximately a third of colorectal and lung cancer diagnoses in the USA have missed diagnostic opportunities despite ‘red flag’ symptoms (eg, rectal bleeding).^5 6^ In England, one in five patients with colorectal cancer diagnosed after an emergency presentation had typical ‘alarm’ symptoms in the year leading up to their diagnosis, and 16%–22% had three or more consultations in primary care with relevant symptoms, suggesting potential opportunities for earlier diagnosis.^7 8^ This points to the importance of understanding what happens within the consultation to prevent patient harm resulting from missed or delayed diagnosis.^9^

Evidence suggests that missed diagnoses^10 11^ are often related to inadequate symptom elicitation^12^ and doctors’ elicitation and interpretation of the presenting problem is critical for subsequent referral decisions.^13 14^ However, elicitation can often be incomplete,^15 16^ and if doctors do not ask for information, patients often do not provide it.^17 18^ Despite a vast existing body of research exploring the conversation between patients and doctors,^19^ which is embedded within doctor communication training (eg, the Calgary-Cambridge model),^20^ there is a lack of in-depth research on doctor-patient interactions to elucidate the essential behavioural factors central to this diagnostic phase.

To date, the focus has been on the role of doctors' communication at the beginning^21 22^ or end of consultations.^23 24^ This has led to important insights, for example, emphasising that interrupting a patient’s opening statement can inhibit elicitation of the patient’s full agenda^21 25^ as well as the importance of concordance, where patients and doctors seek to reach a shared understanding during the consultation.^26 27^

A growing qualitative evidence-base shows that doctor-patient interactions can influence patients’ perceived eligibility/desire for accessing/reaccessing healthcare,^28–30^ but this has focused on asking patients about their experiences of accessing healthcare and has not included healthcare professional views. Patient disengagement (eg, adherence to management plans)^31^ is one of the only patient behaviours highlighted as a potential reason for diagnostic error in primary care.^32^ There are still crucial gaps in knowledge about the unfolding dynamic nature of the diagnostic process, including the patient’s role in the conversation, and how this impacts patients’ subsequent behaviour.^31^

To address these gaps, we used qualitative methods, including video observation triangulated with follow-up interview data from patients and doctors, to understand doctor-patient communication about a new or persistent presenting problem as a critical point in primary care and assessed the potential impact on timely cancer diagnosis. In particular, we examined what was discussed in conversations about new or persistent presenting problems and how doctors and patients negotiated their significance. We also examined how these negotiations impacted patient attitudes and behaviours and considered the possible impact on timely cancer diagnosis.

## Methods

### Research design

We collected data from videos of patient-doctor consultations and related follow-up interviews. Two lay members were recruited to the research team at the development stage of the proposal and contributed to every stage of the research process. Lay members helped focus the research question and study design, obtain ethical approvals (eg, writing patient information sheets), interpret the results and coauthor the manuscript.

Data collection took place in England from July 2017 to March 2018. Primary care practices were recruited with the help of Clinical Research Networks in North London, South London and Surrey. Ethical approval was obtained from London Chelsea Research Ethics Committee (17/LO/0270).

### Recruitment and procedures

Primary care practices were recruited and consenting doctors completed a short demographic questionnaire and indicated whether they agreed to being contacted for a follow-up interview about one of their consultation videos. Following agreement from individual doctors, one researcher (DA) identified and approached eligible patients of these doctors in the waiting area. All patients≥50 years who were able to provide written informed consent were eligible. We focused on adults≥50 years because of their higher risk of cancer.^33^ The researcher obtained written consent, demographic data, the purpose of their consultation and whether they presented with a new problem. Patients also indicated their willingness to be contacted for a follow-up interview. We were particularly interested in patients presenting with any new problem or persistent problem relevant to cancer to maximise the relevance to timely diagnosis. We did not restrict our sample to cancer ‘alarm’ symptoms because there are a large number of symptoms that could be associated with cancer. A new problem was defined as an issue the patient discussed with their doctor for the first time and for which no diagnosis had been formulated yet. This was gleaned from watching all the videos because patients’ self-report of a new problem did not always correspond to this definition. Persistent symptoms were symptoms that patients reconsulted for (without assuming any time threshold) and identified through verbal (eg, the patient saying the problem is not going away) or contextual reference (eg, where the problem is still present after initial treatments or tests). Types of symptoms were taken from NICE guidance for suspected cancer referral,^34^ by searching the word ‘persistent’ within the online NICE guidance tool and matching them with presenting problems in our sample (eg, persistent cold symptoms).

A subset of patients were interviewed about their consultation experiences as well as each doctor about one specific consultation with an interviewed patient. Patients were selected for interview if (a) cancer was explicitly discussed during the consultation and/or (b) the consultation included some level of discussion/uncertainty about the symptom. Patients were selected to avoid over-representing or under-representing any particular doctor. Among consultations for which a patient interview had been conducted, consultations were selected for a doctor interview based on the same criteria (a and/or b). Patients and doctors were interviewed approximately 1 month and 2.5 months after the consultation, respectively. All interviews were carried out by one member of the research team (DA). Interviews were semistructured with an open first part following the Wengraf biographical interviewing method,^35^ which allows participants to tell an uninterrupted story. See online supplementary appendix A for patient and doctor interview schedules.

10.1136/bmjqs-2019-009485.supp1 Supplementary data

### Data analysis

All videos and interviews were recorded and transcribed verbatim. A subset of 40 consultations were coded by DA to include behavioural descriptions such as body language. Each video was watched numerous times during transcription by DA. A research assistant (FA) also transcribed and watched a number of videos to check transcript accuracy. Data were analysed using a thematic analysis approach,^36^ with initial inductive coding developed by DA and then discussed and developed iteratively with the wider research team over the course of 6 months. This wider team included primary care doctors, patient safety experts, psychologists, a public health consultant and two lay members. When selecting patients with a new or persistent problem, DA consulted primary care doctors to validate ambiguous decisions. Where follow-up interview data were available, these were triangulated with video data to further substantiate overall themes.

### Public involvement

Early findings were presented to an advisory group including public representatives, clinicians and academics. The advisory group helped refine initial themes into more flexible dimensions such as cognitive and emotional categories of misalignment. We also ran two focus groups with additional members of the public (n=7 in each group) to discuss the interpretation of the findings and provide further refinement. Suggestions were taken into account and are reflected in this paper.

## Results

### Sample

**Figure 1:**
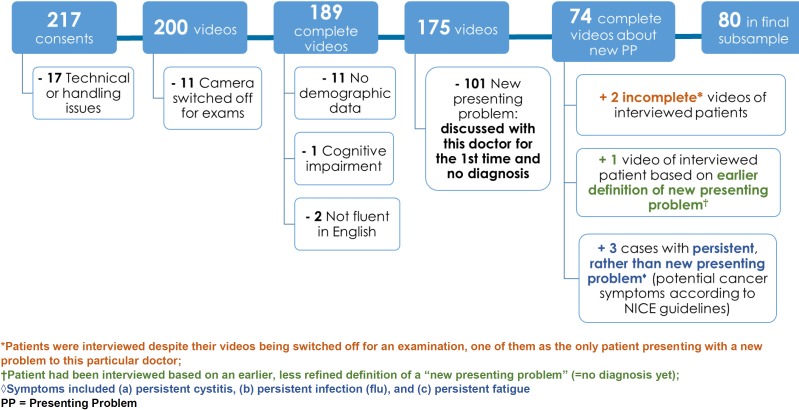
Sample flowchart.

Seven practices agreed to participate in the video observation study and 10 primary care doctors from these practices gave informed consent to participate in video observation. Practices varied in terms of the socioeconomic and ethnicity profile of patients (see online supplementary appendix B for practice characteristics). Of 294 eligible patients approached in waiting areas, 217 patients provided written informed consent (74%). Figure 1 illustrates the subsequent flow of the sample. The final sample (n=80) included those presenting with a new or persistent problem. Of 33 patients who were approached for a follow-up interview, 20 patients (61%) consented. For 7 of these 20 consultations with patient interviews, we additionally interviewed the doctor about the consultation (one doctor per practice). Our final dataset for analysis included 80 videos, 20 patient interviews and 7 doctor interviews. Patient and doctor characteristics are presented in table 1. See online supplementary appendix C for a full list of presenting problems.

**Table 1:** Patient and doctor sample characteristics

	Patients (n=80)	Patient interviews (n=20)	Doctors (n=10)	Doctor interviews (n=7)
Age (years); mean (SD, min–max)	66.5 (11.3, 50–96)	64.8 (9.9, 51–81)	48.2 (10.2, 32–60)	48.0 (9.4, 32–56)
Gender n (%)				
Male	34 (42.5)	10 (50.0)	7 (70.0)	6 (85.7)
Female	46 (57.5)	10 (50.0)	3 (30.0)	1 (14.3)
Ethnic origin n (%)				
White	67 (83.8)	18 (90.0)	9 (90.0)	6 (85.7)
Non-white	12 (15.0)	2 (10.0)	1 (10.0)	1 (14.3)
Prefer not to say	1 (1.2)	–	–	–
Highest education qualification				
Degree or higher degree	18 (22.5)	6 (30.0)	NA	NA
Below degree level	48 (60.0)	10 (50.0)	NA	NA
No formal qualification	14 (17.5)	3 (15.0)	NA	NA
Years since accreditation as a doctor; Mean (SD, min–max)	NA	NA	15.90 (11.5, 2–32)	16 (9.7, 2–27)
Accredited doctor trainer n (%)	NA	NA	6 (60.0)	5 (71.4)
Consultations videoed before n (%)	NA	NA	9 (90.0)	6 (85.7)

NA, not applicable; SD, Standard Deviation; n, sample size.

### Main findings

Below, we present the findings from the video observation and interview data. First, we present the concept of alignment as a process by which doctors and patients negotiate the significance of new or persistent problems. Next, we describe different types of misalignment—where the patient’s expressed significance of a presenting problem was either higher or lower than the doctor’s expression of significance. Finally, we triangulate video and interview data to illustrate the potential impact of misalignments on timely diagnosis of cancer.

### Negotiating alignment of significance

All the consultations involved some level of negotiation about the significance of the presenting problem. Significance in this context is defined as the severity or risk of a presenting problem with respect to disease or to the amount of concern it was causing the patient in day-to-day life. Doctor and patient views of significance could differ mainly because they defined and, crucially, expressed significance differently—the doctor expressing it in terms of medical guidelines (medical significance) and the patient expressing it in terms of what the presenting problem means to him or her personally (personal significance).

This negotiation of significance leads to the concept of *alignment*—a dynamic state indicating the level of agreement between the doctors’ and patients’ communicated significance of their presenting problem. The example in box 1 demonstrates the presence of alignment.

Box 1 **Example of alignment**

Doctor: ‘…the back of the kneecaps can wear out uhm so if you get a bit of err arthritis there or wearing of that, that cartilage then, then it can stick and jar’

Patient: ‘So it’s what I’m wondering’

Doctor: ‘…which is what I think’s happening uhm’

Patient: ‘Yes, I don’t, I, it just, you know, I’ve had it for some time now and I thought one day I’ll ask you about it’

Doctor: ‘Yes (interrupting) yes ok (turning back to his desk to write something) well let’s have a look at that and just confirm things, it’s best you have an x-ray…uhm.’

Patient: ‘Yes, when I, when I go like that- (lifting her left knee up and down)’

Doctor (briefly looking in her direction): ‘You can feel it yes.’

Patient: ‘Yes.’

(Consultation GPHH270)

We found that potentially detrimental patient behaviours and experiences resulted when the patient’s expressed significance of a presenting problem was misaligned with the doctor’s expression of significance and that this was not resolved in the course of the conversation. Importantly, alignment is the result of how significance is expressed and negotiated in the communication, which may or may not fully reflect the doctor’s or patient’s underlying thoughts about the presenting problem’s significance. For example, if a patient downplays a symptom to avoid a diagnostic procedure, resulting expressions of significance could be misaligned between doctor and patient despite both assessing the presenting problem’s significance in a similar way.

### Types of misalignment

Twenty-five (31%) consultations involved a misalignment, suggesting that this is a common problem. We subjectively judged four of these to have been unresolved at the end of the consultation and three only partly resolved. Misalignments usually became apparent during the consultation, as soon as the doctor expressed their initial impression about the presenting problem’s significance. However, misalignments also shaped the conversation before they were recognised and could occur at any stage during the consultation.

If misalignment was identified and openly negotiated, it could be actively brought to a mutually acceptable outcome. This is exemplified in box 2, where a male patient presented with increased urination at night, giving the context that ‘I just wanted to double-check, my parents both died very young, and I don’t have a history of whether there was any cancer in the family.’ The extract follows a physical examination of the patient’s prostate and some hesitation by both the patient and the doctor to continue down the diagnostic route and order a blood test.

Box 2 **Identification of misalignment**

Patient (talking over doctor): ‘Do you think it’s worth doing that, just I mean it’s been like that eh…?’

Doctor: ‘Eh eh i- […] the main thing we look out for is change, and change in your waterworks, and ehm, you know, as men get older yes, they do get up at night more, that doesn’t mean they all got prostate cancer.’

Patient: ‘No, of course.’

Doctor: ‘ehm eh, ophhh……i- it’s difficult ehm, it’s difficult to get- you do have some symptoms, your eh germination is relatively normal, I’d be happy enough if you didn’t do it.’

Patient: ‘Okay.’

Doctor: …’but equally, if you did want to do it, that’s fine, the difficulty is, if you come…if you- eh- if you do- slightly raised…’

Patient: ‘mhm, mhm’

Doctor:… ‘then we’re kind of obliged to pursue it…’

Patient: ‘Yeah yeah yeah.’

Doctor:… ‘then we end up with a biopsy,…’

Patient: ‘Yeah, yeah, yeah.’

Doctor:… ‘and you know, it’s a whole chain of events…’

Patient : ‘Yeah, yeah yeah.’

Doctor: ‘…ehm and that can do harm,…’

Patient: ‘Yeah.’

Doctor: ‘.ehm but we’re sort of on that train a bit now.’

Patient: ‘Mhm, Okay, okay. I don’t want to waste anybody’s time.’ (both now talking over each other)

Doctor: ‘No no no no, it’s not-’

Patient: ‘cause like I said, it’s been like that for ten years-’

Doctor ‘It’s, gee, sometimes it’s just difficult to make the right decision, I- I wouldn’t lose sleep if you didn’t, but (Patient: “No…no.”) I think it’s reasonable to- to do it…ehm’

Patient: ‘I take your advice. Whatever it is you’

Doctor (talking over patient): ‘Let’s just, let’s just do it…’

Patient:… ‘NHS money…(Patient laughs slightly).’

Doctor: ‘Naah, that’s fine, don’t worry.’

(Consultation GPXM195)

Misalignments vary in degree; some were more obvious and were openly discussed, while others were more subtle and not recognised by the doctor and/or the patient. Resolution was easier when the misalignment was a simple difference in knowledge without (obvious) emotional factors. In the example below, the doctor provided knowledge that reassured the patient and their views on significance were easily realigned:

Doctor: ‘The actual colour of it, yellow, is the colour of dead white blood cells, so it shows that you're body's been fighting the infection, white blood cells are dying, new one’s have been generated, and so the right things are happening in your body, okay?’

Patient: ‘And it's thick as well when it comes out.’

Doctor: ‘Yes, it still can be thick but, as I said, it can clump together in the lungs because the tiny little hairs that should line the lungs and keep everything evenly distributed are not working, and therefore bits of phlegm clump together.’

Patient: ‘Okay.’

(Consultation GPWD122)

However, an emotional misalignment may be harder to address. In the next example (box 3), a female patient tried to downplay her presenting problem, until it becomes apparent that she was doing this to avoid diagnostic procedures as a result of a referral.

Box 3 **Example of emotional misalignment**

Doctor: ‘So to be loose is unusual for you?’

Patient: ‘Ehm, it isn’t these days, really, not very eh …b- yeah it is a bit (Patient is nodding to emphasise), it is a bit. Yeah. And certainly, if I’m very loose you now that is definitely not usual.’

[…]

Doctor: ‘…and you say, passing wind, you’re still losing a little bit then as well.’

Patient: ‘Yes.’

Doctor: ‘Is that still happening?’

Patient: ‘No not really it seems I have actually tried to make myself sort of more regular and disciplined this it does seem to have made a difference so I- yeah I think what I am trying to say is I’m very reluctant to have any operations and procedures unless I absolutely have to’

Doctor: ‘No what I think I think one of the things is (Patient: “Hm.”) to do ehm indeed they will need to look inside and and-

Patient: ‘Yes, I’m not very keen on that (laughing slightly) I’ve had that once to find out about the diverti.’

Doctor: ‘Yes well I think certainly we would really want that cause…’

Patient: ‘Yeah.’

Doctor: ‘but just obviously there is a possibility with this eh eh the change here that the looseness now is interesting is you and [husband’s name] have both had it but his has been settled down has it?’

Patient: ‘Yes, it has.’

Doctor: ‘So if yours hasn’t settled, then…’

Patient: ‘But it has really, it has, really…’

Doctor: ‘Alright, but you are still loose?’

Patient: ‘I’m still on the loose side, yeah.’

Doctor: ‘So ehm that that needs to be just checked out.’

Patient: ‘Okay’

Doctor: ‘and- ehm from the point of view could this be something else going on-’

Patient: ‘Yeah’

Doctor: ‘Could this be something nasty like cancer…’

Patient: ‘Yeah.’

[…]

Patient: ‘I personally don’t really think there’s an urgent need but I think it’s right what you are saying that it needs to be looked at.’

(Consultation GPHH248)

The last excerpt shows that alignment might not be a necessary aim in all cases as doctor and patient might still be able to achieve a mutually acceptable outcome despite misalignment. This is particularly exemplified by the patient’s closing remark. However, these cases might also be particularly prone to the potential creation of ‘false’ agreement for both patient and doctor. The doctor may have had a sense of a resolved patient concern, but have missed an opportunity to reorient their appraisal of the problem, or the significance of the problem to the patient.

### Potential impact on timely diagnosis

We identified patient behaviours with potentially harmful consequences for timely diagnosis that resulted from both cognitive and emotional misalignments. Examples include non-attendance at follow-up appointments, declaring lack of trust in the primary care doctor to help resolve problems and deciding to seek help elsewhere (see table 2). Here, we discuss some examples of potential impacts on timely diagnosis in certain cases where it was possible to triangulate video, doctor and patient interview data (n=7).

**Table 2:** Examples of potential impact on timely diagnosis

Negative consequence	Video data	Patient/doctor interview data
Non-attendance at appointments	Doctor: (While studying pictures of moles at computer screen to compare them with the patient’s mole): ‘So yours is a bit like that (pointing at a specific picture) but it’s with the good contrast is….and the fact they’ve changed a bit’ Patient: ‘I’ve had them for years.’ Doctor (still looking at computer screen): ‘Mhm. It’s just a bit like that one there.’ Patient: ‘Mhm.’ Doctor: ‘… you’ve got to be so careful because…the amount of …which is the one we’re worried about is similar ehm. I wonder it’s the safest thing to do, would be, to refer you to see a skin specialist.’ (doctor looking at patient now) Patient: ‘Mhmh, okay.’ Doctor: ‘Ehm, and if we think it could be (short pause) melanoma (gesturing with his hands), which I’m 99.9% sure it isn’t if we do think it could be, we would have to refer you to under what is called the 2 week rule’ Patient: ‘As you what you did for my …throat’ (gesturing towards her throat) Doctor: (nodding in her direction) ‘Yeah.’ (…)	‘I can’t really afford to be ill, as I say, my husband’s in a care home and I see him every day and he has quite a few medical conditions which means that I can’t afford to have any.’ (Patient) ‘Interestingly, looking at her notes, she never kept the appointment to the skin specialist that I can tell, so that is rather odd, that is rather odd.(…)Bear in mind also she was brave enough to come and see me so I suppose you’d have thought she would have kept that appointment but there is no letter back from the hospital at all so I’m assuming she never went.’ (Doctor)
Loss of trust in doctor	Doctor: ‘Ehm. So it could just be a little inf…cyst that’s in the area (Patient: “Yeah.”) cause where it’s red and tender, that is more likely to just be a little bit of maybe an infection that’s there.’ Patient: ‘Yeah.’ and puts the necklace with her glasses back on. Doctor: ‘Ehm but with anything where there is a breast and there is a lump (again pointing towards her breast with her right hand to emphasise) we do want to be completely sure it’s not anything sinister in any way’ (nods a few times in emphasis) (Consultation GPWD59)	‘She did look at it and she just then referred me to the Breast Clinic, which I didn't feel I needed, I felt it was just an abscess or something(…)I ended up having surgery which I feel I didn't need to have.(…)So that's how I feel and I feel the whole experience was terrible.(…)She just said 'oh yes, but we'll send you to the Breast Clinic', that's it,(…)she thought it was probably only a little cyst or an abscess, but she said it's best off to go there. Why, I don't know.(…)It was so red and so angry it must have come to her 'ooh, that's not a cancerous lump' or something, I don't know, unless she thought it was.(…)And I've never seen Dr(doctor’s name)before and I don't wish to see her again at the surgery, so that's how I feel about her.’ (Patient)
Decision to seek help elsewhere	Doctor: ‘Is it falling out or just not growing well?’ Patient: ‘Well it’s a bit of both, it’s a little bit for, you know, you know when you comb your hair, it just seems more than usual, I’m not sure how I can measure that, but it just seems…’ Doctor: ‘Ok yeah.’ (…) Patient: ‘But it’s also not growing- not growing at all.’ Doctor: ‘But generally speaking there’s no bald patches or anything like that.’ Patient: ‘No, I don’t think so.’ Doctor: ‘Other than that it’s pretty…’ Patient: ‘No I don’t think I’ve noticed any bald patches… because I was just wondered whether it’s any sort of, I don’t know, infection, that’s’ that’s what…you know?’ Doctor: ‘Let me just check your blood pressure and have a look at the back of your head…’ (Consultation GPXM228)	‘I didn’t actually feel the doctor really understood what the issue was for me, and I think because there was no examination I just left feeling well, that was a bit of a waste of time.(…)I wasn’t particularly impressed in terms of the consultation and the communication because I don’t think he fully understood the impact you now, everything was having on me really(…)I’ve totally given up on the doctor because I don’t think they understand at all, so I’m actually going to, what am I going, I’m going to see, what have they got, a trichologist we’ve got someone who deals with health, hair and scalp.’ (Patient)

One patient attributed very low significance to a long-standing skin symptom. The patient was referred for suspected cancer (melanoma) on an urgent basis; however, to the doctor’s surprise, she did not keep her appointment. Interview data with the patient revealed that the non-attendance may have resulted from a competing caring responsibility that was not discussed at the consultation.

In another case, the referral decision (for a breast change) was not clearly communicated, as cancer was not mentioned as a possibility (rather the doctor said they wanted to make sure ‘it’s not anything sinister’) and thus their view of the severity of the problem was not shared with the patient. The patient reported feeling confused in the interview and only remembered the mention of a benign abscess being a possibility, leading to a loss of trust in the doctor. Greater transparency may have helped the patient understand the doctor’s concerns.

Finally, a female patient presented with persistent hair loss and headache, which had high personal significance, whereas medical tests did not reveal anything concerning. The consultation did not lead to a mutually acceptable result as the doctor did not take the issue any further (eg, with diagnostic tests or a referral). During the interview, the patient emphasised that her scalp had not been examined, when in fact, the video shows an examination of her scalp. The unresolved discrepancy between the patient’s and doctor’s view of the significance of presenting problem appears to have left the patient with an overall negative impression of the consultation, which affected which details of the conversation were remembered, as well as the patient’s behaviour. In this case, the patient said she would not return to the doctor and would seek help elsewhere.

These examples illustrate how unacknowledged differences between the doctor and patient in their perception of the significance of the patient’s presenting problem can influence patient behaviour and experience.

## Discussion

We found evidence that the doctor-patient conversation could have a detrimental impact on patients’ perceptions and behaviour postconsultation, including non-attendance at follow-up appointments, lack of trust in the doctor and a decision not to reconsult if symptoms persisted. These behaviours were observed in consultations where the doctor’s and patient’s views on the presenting problem were misaligned. Misalignment could happen at any time in the consultation, and could be largely cognitive (eg, differences in medical knowledge) or emotional (eg, different emotional perspectives). Misalignments varied in how easily they were recognised/resolved, with alignments based on differences in knowledge easier to manage than those based on differences in emotional perspectives.

### Comparison with existing literature

The idea that a successful consultation requires negotiation between the patient’s and doctor’s theory of the presenting problem is central across consultation models.^20^ A widely used model in doctor communication training is the Calgary-Cambridge model, which aims to enhance a patient’s involvement in the consultation by enquiring about their ideas, concerns and expectations.^37^ One potential explanation for why emotional misalignment is harder to recognise and resolve in our study than cognitive misalignment could be that eliciting ‘ideas’ (ie, patients’ hypotheses about what is wrong) is more straightforward than eliciting patients’ underlying concerns and expectations. Evidence suggests that doctors’ awareness of patients’ health beliefs differ significantly from patients’ actual beliefs and falsely believe that patients’ beliefs are aligned with their own.^38^ Street *et al* suggested that strategies for increasing awareness could include preconsultation assessment of patients’ beliefs. This suggestion also came up as a recommendation while we discussed our findings with our advisory group. A recent review emphasised that there are currently no interventions that involve engaging patients in the cancer diagnostic interval, and this should be a key focus of future research.^31^

Our findings also support theoretical and empirical research reported in the field of linguistics. For example, in rapport management theory,^39^ Spencer-Oatey describes how rapport can be damaged when behavioural expectations are not met and problematic communication can lead to emotions of sadness and anger.^40^ From the participant interview data, we provide important evidence for this emotional impact, resulting in lack of trust and reluctance to reconsult with the same doctor, and even biased memory as to what happened during the consultation. Our findings thus have practical utility and could be applied to extend existing consultation models to help reduce the likelihood of missed diagnostic opportunities in primary care. For example, although the Calgary-Cambridge model recognises two parallel agendas (patients’ vs doctors’) it does not consider how the presence of these different agendas may result in behaviour that puts the patient at risk of potential harm.

In certain cases, continuity of care seemed to be detrimental to the elicitation process because doctors made assumptions about how their patient would act (eg, in the case of the referral for suspected melanoma) and did not seek information about how their patient’s expectations had changed. Previous research has also shown potentially negative consequences of continuity of care. For example, higher levels of continuity of care have been associated with lower use of referrals or investigations,^41^ possibly due to doctors attributing symptoms to previous morbidity. It is important to recognise the potential impact on timely diagnosis against a backdrop where continuity of care is widely valued by patients.^42^ Previous research has also emphasised the importance of eliciting patient contextual factors (eg, competing responsibilities).^43^ A key finding is that contextual factors elicited actively by a doctor are more likely to be incorporated into follow-up plans than factors revealed spontaneously by the patient,^44^ providing further impetus to elicit patient expectations as early as possible in the diagnostic process.

This concept of alignment is somewhat similar to that of concordance, which refers to doctors and patients aiming to reach a shared understanding about the reason for the consultation, the diagnosis as well as the treatment decision.^26 27^ The notion of concordance is inherent in existing consultation models where the ultimate goal is to reach a shared understanding and develop a mutually acceptable plan. However, in the present study, we introduce *alignment* between doctors’ and patients’ views of significance as a new related concept. Alignment is distinctly unique because (1) it is dynamic and thus ebbs and flows while being negotiated within the consultation (rather than simply an outcome at the end) and (2) has cognitive and emotional facets which may go unrecognised, with important consequences for the diagnostic process. Thus, we take up a process perspective by demonstrating that it may not always be necessary to achieve alignment (an outcome perspective) but to work with the misalignment in a way that facilitates an optimal outcome. In other words, doctor and patient might still not agree about the significance of an issue but it is still possible that they can find a compromise about how to proceed. In contrast to this, an optimal outcome from the perspective of concordance theory appears to be the presence of concordance. We also provide evidence for how unresolved and/or unrecognised misalignment impacts the patient’s behaviour, and possibly even memory, and suggest that consultation models further study this in relationship to diagnosis in order to reduce adverse outcomes.

### Strengths and limitations

A major strength of this study is the triangulation of video observation data with patient and doctor interviews. This rich dataset allowed us to capture multiple views of an interaction as well as verbal and non-verbal communications. Public involvement in this study influenced several aspects, from the initial conception of the research design, methodological approach and interpretation of the findings.^45^

It was not possible to quantify how many cases demonstrated potential impact on timely diagnosis. This is because not all patients were interviewed after the consultation, so we do not know whether they needed further clinical contact. There may also be challenges that develop over time, such as eroded trust in the doctor. However, in the small number of cases (n=7) where we had multiple data sources, there were three instances where potential impacts to timely diagnosis were identified, indicating that it is not a rare occurrence.

Similarly, the number of identified cases of misalignment (regardless of their impact on timely diagnosis) can only be viewed as a rough estimation as identification was largely based on the interpretation of videoed consultations, while some misalignments became more obvious where triangulation was possible. This is an indication that misalignment might be even more prevalent than our data suggest. At the same time, interviewees’ accuracy of recounts might have been hampered by the length of time between consultation and interviews. However, doctors referred to their notes, where necessary, and both doctors and patients appeared able to remember their overall impression of the experience, thus providing us with useful information on longer-term effects of consultations on patient behaviour.

We did not focus on the role of individual differences (gender, ethnicity, education) on the consultation despite previous research recognising its importance.^46–48^ This is beyond the scope of the current paper, although we have amassed a hugely valuable dataset that can be used in future work to tackle this and other pertinent questions.

### Implications

As we included all consultations about new presenting problems rather than focusing on cancer-specific symptoms, we were able to capture the fundamental communication dynamics between doctor and patient in primary care, with broader implications for studying the role of doctor-patient communication for patient safety issues which are not exclusive to cancer. For example, we have highlighted the potential for doctors to be misled by ‘false’ agreement at the end of the consultation while losing opportunities to reorient patients’ concerns during the discussion. However, we recognise that this approach also reduces the cancer-specific relevance of our findings. Future research is required to explore a wider range of consultations where cancer-specific symptoms are discussed as well as provide a larger scale quantification, including reasons for misalignment from doctors and patients. Our findings also have practical implications for primary care. In line with Street *et al*, systems or tools that preassess patients’ beliefs and expectations may be valuable to sensitise the doctor and enhance patients’ confidence.^38^

Longer appointment times for complex cases (eg, where patients present with multiple physical, psychological and social problems) may resolve some misalignments, as well as ensuring patients feel empowered after the consultation.^49^ This may bring benefits in terms of addressing potential cognitive biases associated with continuity of care.^41^ Importantly, in the patient safety literature, the issue of continuity extends beyond seeing the same practitioner, to other potential gaps or ‘discontinuities in care’.^50^ For example, the loss of a follow-up plan (seen in our data when a patient did not attend their specialist appointment) or loss of information may also be seen as gaps in care.

There are also specific cancer-related implications, such as the need for optimal communication at the point of suspected cancer referral, which would align doctor and patient expectations about the need for, and outcome of, the referral. This could be an extension of existing patient safety practices, such as making appointments ahead and discussing what to do if symptoms change or worsen while waiting for the referral appointment. Finally, these issues could be integrated into cancer education tools for primary care.

## Conclusion

Our findings suggest that the doctor-patient conversation in primary care can impact how patients behave after a consultation, with potentially harmful consequences for timely diagnosis. With increasing demands and expectations related to primary care, particularly in terms of diagnosing cancer as early as possible, our findings highlight the complexity of diagnosis-related aspects of the doctor-patient consultation. By highlighting gaps with potential to cause patient harm (eg, eliciting expectations), our findings can be applied to augment their anticipation, detection and bridging and ultimately lead to improving patient outcomes.
