# Supplementary material for: Influence of doctor-patient conversations on behaviours of patients presenting to primary care with new or persistent symptoms: a video observation study
Source: BMJ Qual Saf. 2019 Jul 20;29(3):198–208. doi: 10.1136/bmjqs-2019-009485 (PMC7057803; doi:10.1136/bmjqs-2019-009485)
Supplement: Supplementary data [file bmjqs-2019-009485supp001.pdf]

Appendix A Topic guides for project "**Using qualitative methods to understand the GP/patient conversation"**

[Introduce yourself – check for questions based on PIS – check confidentiality understanding – take formal consent (both sign) – check about audio recording]

- *trying to understand how GPs and patients communicate with each other*
- *need to know how the conversations go*
- *also interested in how consultations lead to a referral to a hospital*

We're going to start by talking about how you decided to come and see the GP.

Tell me the story of how you came to see the GP recently (Patient)

So please tell me the story of the consultation, just in your own words (GP)

- *All the experiences and the events which were important for you, up to **now***
- *Start wherever you like*
- *Please take the time you need*
- *I'll listen first, I won't interrupt*
- *I'll just take some notes in case I have any further questions for after you've finished telling me about it all*

Non-specific prompts:

Are there any other things you remember happening?

Does it make you think of anything else that has happened?

Are you thinking about something else that happened?

WITHOUT specifying the content of what the storying should be about, of those 'other things', the 'anything else'.

Specific prompts (i.e. if not mentioned already):

What did you tell the GP/patient?

What did the GP/patient ask you about?

What did you/they advise them/you to do? (GP)

What did you think of that?

What's been happening more recently?

Appendix B Characteristics of the seven general practices in the sample

Practice	List size (n)	Deprivation index	Ethnicity (mixed) (%)	Ethnicity (Asian) (%)	Ethnicity (Black) (%)	Ethnicity (Other non-white) (%)
**1***	15093	10	1.1	1.7	0	0
**2***	6744	5	6.3	6.8	6.6	2.7
**3***	11642	9	2.1	6.7	1.6	1.6
**4**	7156	3	7.4	5.4	29.6	2.9
**5**	3220	5	5.5	26.1	21.2	2.4
**6**	19176	4	8.1	6.0	25.4	1.9
**7***	23979	10	1.8	3.0	0	1.1

*Note. Deprivation Index from 1-10 with 1= most deprived decile, 10=least deprived decile;*Training practice.*

Appendix C Overview of individual participants (n=80) including their new or persistent presenting problem(s).

Consultation ID	Age	Sex	Symptom
**GPCC7**	78	Female	Mole
**GPCC9**	65	Female	Allergy, vertigo, dizziness, high blood pressure
**GPCC47**	69	Female	Allergy/severe rash
**GPCC39**	69	Male	Pain on right side of belly/shoulder pain
**GPCC23**	54	Female	Persistent fatigue (chronic fatigue/fibromyalgia type of picture)
**GPCC32**	83	Male	Pain in back
**GPPT83**	60	Male	Bruised shin/injury
**GPPT72**	63	Male	Persistent cold symptoms
**GPPT69**	75	Male	Injury at foot
**GPML211**	73	Female	Hip & knee pain after fall
**GPML219**	69	Female	Cough, sore throat
**GPML225**	83	Female	Lump, breathlessness
**GPML227**	82	Female	Breathing difficulty, chest pain
**GPML241**	79	Female	Breaking nails/calcium levels
**GPML242**	53	Female	Persistent sore throat, depressive symptoms
**GPML232**	68	Female	Weight loss, stomach problems
**GPXM204**	54	Male	Difficulty swallowing, heartburn
**GPXM228**	52	Female	Hair loss, headache
**GPXM215**	62	Female	Restricted arm movement
**GPXM195**	50	Male	Prostate issue
**GPXM216**	69	Female	Mark on arm
**GPXM209**	63	Female	Rash, high blood pressure
**GPWC163**	51	Female	Vaginal dryness
**GPWC164**	79	Female	Rattley chest
**GPWC187**	72	Female	Belly and back pain, eye issue
**GPWC166**	51	Male	Persistent cough, leg pains
**GPWC168**	60	Female	Pain in foot
**GPWD51**	51	Male	Itchy wrists

**GPWD61**	57	Male	Ear problems
**GPWD63**	67	Female	Bitter taste in mouth
**GPWD64**	60	Female	Keratin plugs, vaginal dryness
**GPWD122**	56	Male	Persistent: cough/chest infection; new: sprained muscle
**GPWD127**	63	Male	Mole
**GPWD128**	72	Female	Cystitis
**GPWD137**	88	Male	Sore throat, vision problems
**GPWD144**	50	Male	Depression
**GPWD145**	64	Female	Muffled hearing
**GPWD149**	52	Female	Cystitis
**GPWD154**	64	Female	Persistent cold
**GPWD155**	68	Female	Leg pain, vaginal dryness
**GPWD157**	62	Female	Pain in foot
**GPWD158**	51	Male	Difficulty swallowing, acid reflux
**GPWD159**	88	Female	Anxiety
**GPWD161**	67	Female	Lung problems
**GPWD171**	50	Male	Persistent cough
**GPWD176**	75	Male	Diarrhoea
**GPWD180**	63	Female	Recurring cystitis
**GPWD181**	70	Male	Pain in chest, shoulder pain, numbness
**GPWD182**	54	Female	Knee injury
**GPWD183**	55	Female	Pain in toe
**GPWD186**	78	Female	Blister on leg
**GPWD189**	68	Female	Symptoms of urinary tract infection, work stress, low white blood cell count
**GPWD190**	88	Male	Coldness sensation in hand
**GPWD191**	52	Female	Rash, shoulder pain
**GPWD198**	54	Female	Menopausal symptoms
**GPWD200**	72	Female	Digestive issues, acid reflux
**GPWD160**	60	Female	Constipation

**GPWD170**	72	Male	Swelling feet, leg cramps
**GPWD153**	52	Female	Itchy feet, suicidal tendencies
**GPWD124**	53	Female	Painful toenail after injury
**GPWD66**	70	Male	Soreness between buttock cheeks
**GPWD59**	59	Female	Breast lump
**GPPTU143**	78	Male	Hand injury, (dizzy spells, high calcium levels)
**GPPTU140**	71	Male	Allergic skin reaction
**GPPTU142**	60	Male	Injured foot, bulging veins, pain in hands, moles, hearing difficulty
**GPBP239**	58	Male	Numbness in foot and leg
**GPBP293**	96	Female	Pain in hand
**GPBP295**	76	Male	Tummy problems, headache
**GPBP297**	86	Male	Persistent severe nausea
**GPHH264**	61	Male	Bleeding gums, reflux, unclarified irregularity on x-ray
**GPHH259**	74	Female	Arm movement pain
**GPHH255**	87	Male	Diarrhoea, frequent urination
**GPHH270**	81	Female	Knee pain
**GPHH279**	65	Male	Vision problems
**GPHH282**	72	Male	Painful knees, itchiness, lump in groin
**GPHH284**	74	Male	Moles on face and on scalp, joint pain
**GPHH269**	71	Male	Tinnitus, nausea, headache
**GPHH272**	52	Female	Recurring infection (flu like symptoms)
**GPHH248**	70	Female	Persistent bowel problems
**GPHH281**	74	Male	Mole, shoulder pain
